# Personalized hematopoietic stem cell transplantation for inborn errors of immunity

**DOI:** 10.3389/fimmu.2023.1162605

**Published:** 2023-04-05

**Authors:** Mary Slatter, Su Han Lum

**Affiliations:** ^1^ Paediatric Immunology and HSCT, Newcastle University, Newcastle upon Tyne, United Kingdom; ^2^ Translational and Clinical Research Institute, Great North Children’s Hospital, Newcastle upon Tyne, United Kingdom

**Keywords:** hematopoietic stem cell transplantation, inborn errors of immunity, SCID-severe combined immunodeficiency, conditioning, T cell depletion

## Abstract

Patients with inborn errors of immunity (IEI) have been transplanted for more than 50 years. Many long-term survivors have ongoing medical issues showing the need for further improvements in how hematopoietic stem cell transplantation (HSCT) is performed if patients in the future are to have a normal quality of life. Precise genetic diagnosis enables early treatment before recurrent infection, autoimmunity and organ impairment occur. Newborn screening for severe combined immunodeficiency (SCID) is established in many countries. For newly described disorders the decision to transplant is not straight-forward. Specific biologic therapies are effective for some diseases and can be used as a bridge to HSCT to improve outcome. Developments in reduced toxicity conditioning and methods of T-cell depletion for mismatched donors have made transplant an option for all eligible patients. Further refinements in conditioning plus precise graft composition and additional cellular therapy are emerging as techniques to personalize the approach to HSCT for each patient

## Graphical Abstract

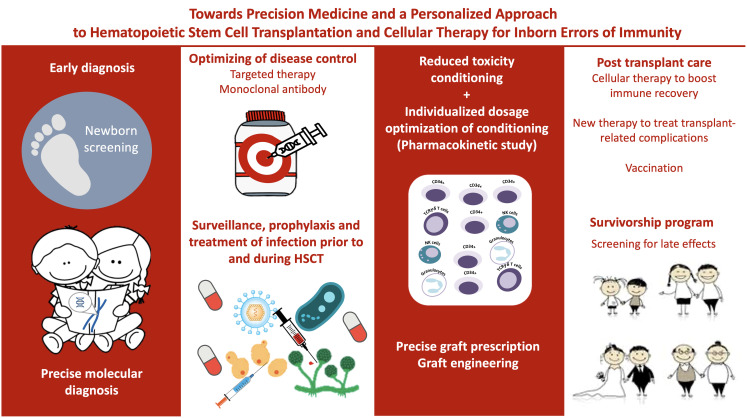

## Introduction

The first allogeneic hematopoietic stem cell transplants (HSCTs) were carried out in patients with X-linked severe combined immunodeficiency (SCID) and Wiskott Aldrich syndrome (WAS) in 1968 (1–3). Nowadays the number of patients and range of inborn errors of immunity (IEI) for which HSCT is indicated, and may be the treatment of choice has expanded exponentially. The arrival of genomic testing has made it possible to make a precise molecular diagnosis at an early stage, which helps to guide optimal treatment. More than 450 single gene defects have been identified and are classified according to an international committee of experts (4). With earlier recognition of disease, patients may be considered for transplant before they have organ impairment and their condition can be optimized by treating infections, autoimmunity or other comorbidities, leading to improved outcomes. Increased use of targeted sub-myeloablative or low toxicity myeloablative conditioning regimens, improved methods of graft manipulation, and better ways of treating post-transplant complications have also led to a dramatic increase in survival. However, many patients that were transplanted 20 to 30 years ago have ongoing medical issues and it is important to continue to optimize approaches to transplant in order that long-term toxicities may be avoided in the future to ensure patients have a good quality of life (5, 6).

This article will review some of the new approaches in order to achieve the goals of transplant: to limit graft failure and graft versus host disease (GVHD), limit short and long-term toxicity, promote long-term good immune reconstitution and quality of life.

## Indications

Historically, HSCT for SCID has led to a higher survival than for non-SCID IEI. In Europe, data from participating transplant centers are collected in the Stem Cell Transplantation for Immunodeficiencies in Europe (SCETIDE) registry, which report progressive improvement in survival over time (7–12).

## Severe combined immunodeficiency

SCID is the most profound IEI affecting cellular and humoral immunity. New guidelines published by the Primary Immune deficiency treatment consortium in the USA define patients with typical SCID as having less than 0.05 × 10^9^ autologous T lymphocytes per litre on repeated tests, with either pathogenic variant(s) in a SCID-associated gene, very low or undetectable T-cell receptor excision circles or less than 20% of CD4 T cells expressing naive markers, and/or transplacental maternally engrafted T cells. Patients with less severely impaired autologous T-cell differentiation are categorized as having leaky or atypical SCID, with 2 or more of the following: low T-cell numbers, oligoclonal T cells, low T-cell receptor excision circles, and less than 20% of CD4 T cells expressing naive markers. These patients must also have either pathogenic variant(s) in a SCID-associated gene or reduced T-cell proliferation to certain mitogens. For a definition of Omenn syndrome patients should have a generalized erythematous rash, absent transplacentally acquired maternal engraftment, and 2 or more of the following: eosinophilia, elevated IgE, lymphadenopathy, hepatosplenomegaly (13). More than 20 genes responsible for different types of SCID have been identified (14). SCID presents in infancy with persistent and opportunistic infections and failure to thrive and when left untreated, infants usually die within the first year of life (15). Thousands of infants have undergone HSCT for SCID around the world. Survival has progressively improved with time and importantly, patients diagnosed and transplanted before the development of infection have a better outcome which has led to the introduction of newborn screening in many countries (16–22). The main challenge, once diagnosed, is how to keep patients free from infection until definitive treatment. In a recent report, despite diagnosis following newborn screening, some patients developed infections prior to HSCT which contributed to the surprising lack of difference in overall survival (OS) between patients diagnosed clinically compared with those diagnosed *via* newborn screening or family history (23). This emphasizes the need for evidence-based guidelines for managing these infants pre- HSCT (24).

Gene therapy was pioneered for adenosine deaminase-deficient (ADA) SCID, followed by X-linked SCID, in the 1990s. Autologous hematopoietic stem cells were transduced with long terminal repeat – intact gammaretroviral vectors and transplanted (25, 26). The vectors now used in trials have been redesigned because the initial trials in France and England were complicated by vector-related T-lymphocyte leukaemia (27, 28). More than 150 patients have been treated with gene therapy for ADA deficiency in various trials (29, 30). Gene therapy has also been successful for patients with other IEI such as WAS (31) and trials are in progress for Recombinant activating gene (RAG)1 deficiency and Artemis deficient SCID (32, 33). More precise genetic manipulation of hematopoietic stem cells by gene editing is also being studied (34).

As survival from treatment has improved so much, more information is available on the long-term outcome of patients treated for SCID (35–39). Poor immune reconstitution leads to problems such as infection and autoimmunity. Some patients suffer from chronic GVHD which causes morbidity and poor quality of life. Chemotherapy can also cause long-term organ impairment and lead to infertility. Therefore the way in which we transplant patients today has an important influence on not just their survival, but their quality of life to come. The aim is to produce minimal short- and long-term toxicity with normal immune reconstitution.

Historically many transplants for SCID were performed without pre-transplant conditioning with chemotherapy, especially if a genoidentical/matched sibling donor (MSD) was available. We now recognize that, even for T-B + NK- SCID phenotypes, although full myeloid chimerism is not necessary for cure, a degree of myeloid engraftment will enable B-lymphocyte reconstitution and long-term thymopoiesis (37, 38). Lankester et al. recently reported that in 338 genetically defined SCID patients in the SCETIDE registry, transplanted between 2006 and 2014, there was no significant difference in OS between those who received conditioning and those who did not. Importantly it was reported that the likelihood of achieving a naïve CD4+ count of >500 cells/μL and attaining immunoglobulin independence was associated with conditioning in all genotypes (12). The latest ESID/EBMT Inborn Errors Working Party (IEWP) guidelines currently recommend conditioning for all genetic types of SCID, no matter which donor is available, as the default setting in most cases. The clinical status of the patient needs to be taken into account. If there is life-threatening infection, the patient could not tolerate chemotherapy, and there is an available matched family donor (MFD), an unmanipulated T-lymphocyte replete graft infusion can provide functional T-lymphocytes from the donor which proliferate and expand in the recipient quickly to control infections. In such cases a second, conditioned procedure may be planned once infection has resolved, depending on the SCID genotype and if there is evidence of incomplete immune reconstitution (40).

Key concepts for SCID:

- Newborn screening is now established in many countries
- Prevention of infection prior to definitive treatment affects outcome
- Conditioning prior to HSCT is recommended for all, to ensure good long-term outcomes

## Emerging IEI

Patients with undefined T cell deficiencies historically had a worse outcome which maybe because transplant only took place as a last resort (11). A precise genetic diagnosis can lead to targeted therapy and it is expected that increasingly with time patients transplanted with a genetic diagnosis, rather than having a retrospective genetic finding, will have better outcomes. The San Francisco center reported 98 patients transplanted for IEI from 2007 to 2018. At the time of HSCT the underlying genetic condition was known in 85% of cases. Both event-free survival (EFS) and OS at 5 years were superior for patients with a known genetic diagnosis (78% with versus 44% without, *p* = 0.006; and 93% versus 60% without, *p* = 0.0002, respectively). OS at 5 years was superior both for patients with SCID and non-SCID IEI if the genetic diagnosis was known. Graft failure occurred less frequently in patients with a known genetic diagnosis (19% with a genetic diagnosis versus 47% without, *p* = 0.020). There was no difference in OS between HSCT performed between 2007 and 2010 compared to more recently (*p* = 0.19). These data show the importance of gene sequencing as standard of care (41).

As more diseases are discovered and elucidated an increasing number of disease specific outcomes have been published.

An example of a relatively recently described disorder is Dedicator of cytokinesis 8 defects (DOCK 8). In 2009 such defects were found to cause abnormal cytoskeletal rearrangement, leading to abnormal cell structure, defective migration and adhesion causing an autosomal recessive combined immunodeficiency. Patients present with viral infections, atopic eczema, impaired T-cell function and TH17 cell differentiation, with defective eosinophil homeostasis and dysregulation of IgE (42, 43). In 2015 Aydin et al. reported 136 patients with DOCK 8 deficiency who had a probability of OS (censored for HSCT) of 87% at 10, 47% at 20, and 33% at 30 years of age, respectively. EFS was 44, 18 and 4% at the same time points with events defined as death, life threatening infections, malignancy or cerebral complications such as CNS vasculitis or stroke. Malignancy was diagnosed in 23/136 (17%) patients (11 haematological and 9 epithelial cancers, 5 other malignancies) at a median age of 12 years. Eight of these patients died from cancer. Severe, life threatening infections were observed in 79/136 (58%); severe non-infectious cerebral events occurred in 14/136 (10%). This report demonstrated the severity of the disease with relatively poor prognosis and recommended early HSCT as a potential curative measure (44). Eighty-one patients were subsequently reported from 22 centers who had received HSCT for DOCK 8 deficiency at a median age of 9.7 years (range, 0.7-27.2 years) between 1995 and 2015. After a median follow-up of 26 months (range, 3-135 months), 68 (84%) patients were alive. Reduced-toxicity conditioning based on either treosulfan or reduced-dose busulfan led to superior survival compared with fully myeloablative busulfan-based regimens (97% vs 78%; *p* = .049). Ninety-six percent of patients younger than 8 years at HSCT survived, compared with 78% of those 8 years and older (*p* = .06) demonstrating the importance of transplant at a younger age before organ impairment (45).

Another newly described IEI is cytotoxic T lymphocyte antigen 4 (CTLA-4) haploinsufficiency. Pathogenic mutations in CTLA-4 behave in an autosomal-dominant manner with incomplete penetrance, leading to an immune dysregulation syndrome with impaired T- and B-cell homeostasis. In 2014 Kuehn et al. identified 7 patients from 4 families with lymphoproliferation, organ infiltration, autoimmune cytopenias, and B-cell abnormalities (46). In the same year Schubert et al. identified 14 patients from 6 families, of whom 11 had enteropathy and 10 hypogammaglobulinemia; other manifestations included granulomatous lymphocytic interstitial lung disease (ILD), respiratory infections, organ infiltration, cytopenias, lymphadenopathy, skin diseases, autoimmune thyroiditis, arthritis, and one case of solid cancer (47). The CTLA-4 fusion proteins Abatacept and Belatacept have shown promising results in ameliorating disease symptoms, but the very long-term outcome of their use is unknown (48, 49).

Fifteen patients are reported who have undergone HSCT for CTLA-4 haploinsufficiency (50, 51). In the first report 6 out of 8 transplanted patients survived, but one death was due to diabetic ketoacidosis 2 years post transplant rather than transplant related mortality (TRM) (50). In the second report 9 of 12 patients survived HSCT (3 also reported in the 1st series) (51). In all cases problems such as cytopenias and enteropathy resolved, but organ impairment such as diabetes cannot be reversed.

A further example is signal transducer and activator of transcription (STAT) 3 gain-of-function (GOF) syndrome, which was recently recognized as an autosomal dominant primary immune regulatory disorder (PIRD) (52). Patients have early-onset autoimmunity, lymphoproliferation, susceptibility to infection, and growth failure. The immune phenotype of STAT3 GOF patients is variable, but hypogammaglobulinemia and lymphopenia are common features along with elevated double-negative (CD4-CD8-) T-cell populations and reduced Treg cells. Treatment is challenging, often requiring multiple immunosuppressive medications. Recently, success with targeted therapy using Janus kinase (JAK) inhibitors and/or anti–IL-6R blockade has been reported (53, 54). An international cohort of 191 affected patients has recently been published which showed that genotype did not predict survival; nor was it revealing as a prognostic indicator of disease manifestations. The constellation of features most common in this disorder (lymphoproliferation, autoimmune cytopenias, enteropathy, ILD, growth failure) are shared by many PIRDs caused by other gene defects [eg, Forkhead box (FOX)P3, CTLA-4, Lipopolysaccharide-responsive beige-like anchor (LRBA), phosphoinositide 3-kinase delta (PIK3CD)] demonstrating the importance of genetic testing. Most patients required 5 or more treatment modalities and targeted therapy led to improved patient outcomes. Twenty-three patients underwent HSCT for treatment-refractory life-threatening disease manifestations, 10 of whom died. Thirteen patients who had not had a HSCT died at a mean age of 23 years (4 months to 52 years) from causes including renal failure, progressive respiratory failure, severe enteropathy, and multiorgan failure (55).

This highlights the dilemma when a molecular diagnosis is made of whether to plan an early HSCT with a better outcome before organ impairment, recurrent infection and possible malignancy occur, treat with targeted therapy for which the long-term effects are unknown, or wait for HSCT when further disease manifestations occur. This is one of the fundamental challenges going forward for many of these more newly described diseases which requires careful patient counselling with rigorous collection of long-term outcome data for the different therapeutic options.

Key concepts for emerging IEI:

- Knowledge of an underlying genetic diagnosis improves outcome of HSCT
- HSCT at a younger age before organ impairment improves outcome of HSCT
- Determining which patients to offer HSCT to is challenging
- Long-term outcome studies are required to compare conservative management, targeted therapy and HSCT outcomes

## Surveillance and treatment of infections

Preparation of patients for transplant is of paramount importance in order to optimize outcome. This includes finding and treating any infections before, during the transplant period and whilst immune reconstitution is established.

Screening for viral infections by PCR allows early treatment for viruses such as cytomegalovirus (CMV) and adenovirus, before disease becomes clinically apparent. Antiviral drugs including cidofovir, ganciclovir and foscarnet have significant side effects. Less toxic agents such as Brincidofovir are not widely available (56). Letermovir inhibits CMV replication and is licensed for CMV prophylaxis, but not in the paediatric population. It interacts with Calcineurin and mTOR inhibitors and azoles, caution is advised in hepatic and renal impairment, but it is relatively well tolerated (57). Maribavir inhibits CMV DNA replication, encapsidation, and nuclear egress of viral capsids *via* inhibition of the UL97 protein kinase. It is useful for the treatment of refractory CMV infection. It has recently been approved for use by the FDA in patients of 12 years or older. Some centers are able to access specific antiviral cytotoxic T-lymphocytes usually on a compassionate basis or as part of a clinical trial. These may be donor-derived or third party banked cells and can be virus or multi-virus specific. Usually they are used post HSCT before immune reconstitution matures, but they can be used pre-transplant in infected patients to try to minimize infective load prior to HSCT (58, 59). A multi-national placebo-controlled phase III tri-viral (CMV, Adenovirus, Epstein Barr virus (EBV))- specific T-lymphocyte clinical trial is currently in progress, based in Munich (TRACE -TRansfer of Adenovirus, CMV and EBV-specific T cells).

Another way of boosting immune reconstitution post HSCT is to use modified donor lymphocyte infusions (DLI) which have a lower risk of causing GVHD than conventional DLI. These include depletion of naïve CD45RA+ T-lymphocytes, which significantly reduces alloreactivity whilst preserving memory CD45RO+ T-lymphocytes with antiviral properties. This method is discussed further in the section on T-lymphocyte depletion.

A number of guidelines exist for the prophylaxis and treatment of fungal infections in transplant recipients and newer agents such as posaconazole and isavuconazole have better safety and tolerability profiles (60–62).

Prophylaxis for pneumocystis jeroveci and bacterial infections is well established.

Key concepts for surveillance and treatment of infections:

- Prophylaxis remains important for patients with IEI particularly going through HSCT
- Screening for infections particularly by PCR for viruses enables pre-emptive treatment
- New agents are available for effective and less toxic treatment for some infections
- Additional cellular therapies are emerging to combat infections

## Bridging treatments

Precision based therapies are becoming increasingly available particularly for some PIRDs. Whilst the long-term use of these biologic modifiers as long-term treatment is unknown, using them to control disease manifestations prior to HSCT is an attractive option (63, 64).

Results of HSCT for patients with Hemophagocytic lymphohistiocytosis (HLH) are poor if patients are not in remission at the time of transplant (65). For many years the first-line treatment has been a combination of etoposide, cyclosporine and dexamethasone with additional intrathecal therapy for CNS disease, according to the HLH 2004 protocol (66). This is associated with significant toxicity particularly from prolonged use of etoposide. Alemtuzumab, a humanized monoclonal anti-CD52 antibody, is currently being studied as first-line therapy with impressive results in more than 50 patients treated with almost 91% survival (67). In combination with methylprednisolone and cyclosporine, alemtuzumab led to a partial response in 64% of treated patients in a previous report (68). The interferon-gamma targeted monoclonal antibody Emapulumab showed promising results in achieving remission (69) and targeting key cytokines such as IL1, IL6 and inhibition of the JAK1/2-STAT1 pathway are also being increasingly used (70–72).

CTLA-4 binds to the CD80 and CD86 ligands on antigen presenting cells outcompeting CD28 mediated activating signals which downregulates the immune response by inhibiting T cell activation. Absence of LRBA leads to decreased CTLA-4 expression, which results in impaired Treg cell function. Loss of expression of LRBA protein due to biallelic mutations in LRBA and CTLA-4 haploinsufficiency therefore share many of the same immunological and clinical features. Sirolimus inhibits the CD28 signalling pathway which decreases T cell hyperactivity. It also enhances T regulatory cells and has been shown to be effective. The CTLA-4 fusion proteins Abatacept and Belatacept show promising results in these disorders (48, 49).

Gain of Function mutations in PIK3CD or loss of function mutations in PIK3RI lead to Activated PI3K delta Syndrome (APDS) (73). Sirolimus inhibits downstream enhanced mTORC1 activity and more recently Leniolisib, an oral small molecule inhibitor of the p100delta subunit of PI3K, was reported to inhibit PI3K activation directly (74).

GOF mutations in STAT1 and STAT3 cause hyperactivation in STAT1 and STAT3 respectively. Jakinibs are direct inhibitors of the JAK/STAT pathway. Ruxolitinib or tofacitinib have been used successfully to target the STAT hyperresponsiveness in patients with STAT1 and STAT3 GOF (53).

Key concepts in bridging therapies:

- Biologic modifiers are available to treat specific diseases
- Outcome of long-term therapy needs to be studied
- Optimising patient status prior to HSCT with such agents is useful to improve outcome

## Conditioning

Historically, conditioning therapy prior to HSCT for patients with IEI was based on the alkylating agents busulfan and cyclophosphamide. Such preparative regimens are associated with significant short and long-term toxicity and a high incidence of TRM. This led to the development of immunosuppressive, less toxic or low intensity regimens with fludarabine and melphalan. This enabled patients with significant co-morbidities to have a much better chance of survival (75, 76). However, mixed chimerism and a high incidence of late viral reactivation can cause problems. Late onset acute GVHD can also occur particularly if DLIs are used to improve chimerism. In adults there are reports of cardiac toxicities associated with the use of melphalan (77, 78) which have not been published in children.

Therefore the use of reduced toxicity regimens is now the preferred choice. The commonest regimens are based on a combination of either treosulfan or busulfan with fludarabine, with additional thiotepa to enhance myeloablation if required.

Treosulfan (*L*-treitol-1, 4-bis-methanesulphonate) is a water-soluble bifunctional alkylating agent, that is related to busulfan with the addition of 2 hydroxyl components. It is a pro-drug, which undergoes pH and temperature-dependent non-enzymatic conversion to its active derivatives spontaneously under physiological conditions. As it is water soluble it can easily be given intravenously. Unlike busulfan it is not metabolized in the liver so hepatic complications, such veno-occlusive disease (VOD) are rarely seen and drug interactions with other medications such as glutathione level reducers (e.g. cyclophosphamide, paracetamol), hepatic enzyme inducers (e.g. itraconazole) and substrates such as methylprednisolone, are fewer. Treosulfan is also significantly less neurotoxic than busulfan as it does not cross the blood brain barrier so no prophylactic anticonvulsant therapy is required. The main toxicities are mucosal, and skin rashes, particularly in the perineal area, which may require pain relief. Most centers recommend frequent bathing and nappy changes in infants receiving treosulfan (79). Early studies reported its use in adults with haematological malignancies considered to be at too high risk for conventional conditioning (80, 81). The first reported use exclusively in children was in 3 patients with Schwachman–Diamond syndrome, who received fludarabine, treosulfan and melphalan. All engrafted, but 1 died post-cord-blood HSCT with idiopathic pneumonitis syndrome (82). Greystoke et al. reported 32 children who were conditioned with treosulfan prior to HSCT for a variety of non-malignant diseases in 2 UK centers including 18 patients with IEI. There was little regimen-related toxicity and the children with IEI did particularly well, with only 1 death in a patient with HLH and 2 with low-level chimerism needing consideration of a second procedure (83). Successive publications have demonstrated safety and efficacy leading to widespread use of treosulfan in conditioning for HSCT for IEI and incorporation into the IEWP of the EBMT and ESID guidelines for HSCT for IEI (40, 84–91). A number of pharmacokinetic (PK) studies have been performed which show variable results. High interindividual variability occurs and several studies show a relationship with increased mucositis and skin toxicity with high treosulfan area under the curve (92–94). Mohanan et al. found a trend to better OS with an area under the concentration curve (AUC) < 1828mg*h/L and that lower treosulfan clearance was significantly associated with OS and EFS (95). Chiesa et al. found that a higher AUC was associated with a higher risk of mortality in multivariable analysis and a trend for low AUC to be associated with poor donor engraftment (myeloid chimerism ≤ 20%) (94). Young children have lower renal clearance and lower central volume of distribution and so dose reduction is required to limit exposure. Whilst all centers tend to reduce the dosing in young infants, some follow the recommended body surface area dosing, whilst others use body weight and age to set the dose parameters and may vary these according to diagnosis. Therapeutic drug monitored-guided dosing should be explored particularly for infants and young children undergoing HSCT.

Long-term outcome using treosulfan is beginning to show encouraging results for the risk of gonadal toxicity. Thirty-five of 56 patients (63%) who received busulfan-based conditioning for non-malignant diseases, in a single center, were found to have gonadal dysfunction compared to 9 of 32 (28%) who received treosulfan-based conditioning. Lower busulfan exposure was not associated with a reduced risk of gonadal dysfunction. The majority of patients in the busulfan group received cyclophosphamide, whilst the treosulfan recipients received thiotepa and fludarabine in the majority of cases (van der Stoep, Personal Communication). Leiper et al. reported serum concentrations of anti-Müllerian hormone (AMH) in females and inhibin B in males in survivors of HSCT in 3 groups: group A received treosulfan-based conditioning, group B, fludarabine and melphalan, and group C, busulfan and cyclophosphamide. Serum AMH and inhibin B were significantly higher in group A compared with groups B and C, suggesting that treosulfan-based regimens have a less damaging impact on gonadal function (96). Faraci et al. reported 137 patients in an EBMT retrospective study of whom 118 received busulfan and 19 treosulfan. A higher proportion of girls in the treosulfan group in the prepubertal stage reached spontaneous puberty compared with those treated with busulfan (*p* = 0.02). Spontaneous menarche was more frequent after treosulfan than after busulfan (*p* < 0.001). Postpubertal boys and girls treated with treosulfan had significantly lower luteinizing hormone levels (*p* = 0.03 and *p* = 0.04, respectively) compared with the busulfan group. Frequency of gonadal damage associated with treosulfan was significantly lower than that observed after busulfan albeit with low numbers in the treosulfan group (97).

Thiotepa is an alkylating agent which is metabolized in the liver to its active metabolite. It is excreted in the urine but also through the skin *via* sweat (98). Despite frequent use of thiotepa in addition to treosulfan and fludarabine, there is limited data comparing this combination to treosulfan and fludarabine alone (88). Reports indicate that the addition of thiotepa does not seem to increase short-term toxicity, but no formal studies have been done. Thiotepa is an alkylating agent and would be expected to have an impact on fertility, so studies of late effects are also needed. Additional thiotepa is usually given for diseases which are more difficult to engraft or require full donor chimerism in all cell lineages. There are no published studies on thiotepa PK in the allogeneic HSCT setting (99).

It is routine practice when using busulfan to do therapeutic monitoring and adjust the doses according the target AUC. The use of a lower target AUC (45–65 mg/L × h) combined with fludarabine was introduced by Tayfun Güngör and colleagues in Zurich. Fifty-six children and young adults with chronic granulomatous disease (CGD) were reported in 2014, Many of them had high-risk features such as intractable infections and autoinflammation. Twenty-one MFD and 35 matched unrelated donor (MUD) transplants were performed. The 2-year probability of OS was 96% (95% CI 86·46–99·09), and of EFS was 91% (79·78–96·17). Graft-failure occurred in 5% (3 of 56) of patients. The cumulative incidence of acute GVHD of grade III–IV was 4% (2 of 56) and of chronic GVHD was 7% (4 of 56). Stable (≥ 90%) myeloid donor chimerism occurred in 52 (93%) surviving patients (100). In the IEWP guidelines 2 alternative protocols are suggested with either a myeloablative (85-95 mg/x h) or reduced toxicity range in combination with fludarabine (40). Dvorak et al. reported the results of the use busulfan at a very low target AUC (30 mg/L × h) alone or in combination with fludarabine or thiotepa in 10 patients with SCID. All the patients survived, 1 required a second HSCT, and 3 had no B cell reconstitution (101).

Bognar et al. recently reported an observational study 697 patients who underwent allogeneic HSCT with intravenous busulfan as part of the conditioning regimen at 15 paediatric transplantation centers between 2000 and 2015. Eighty-eight of 697 patients (12.6%) developed VOD. For patients receiving only busulfan as an alkylator (36.3%, n = 253), cumulative busulfan exposure (>78 mg x h/L) was associated with an approximately threefold increased risk of VOD (12.6% versus 4.7%; odds ratio [OR] = 2.95, 95% confidence interval [CI] 1.13 to 7.66). For patients receiving busulfan with one or two additional alkylators (63.7%, n = 444), cumulative busulfan exposure (<78 and >78 mg x h/L) did not further increase the risk of VOD (15.4% versus 15.2%; OR = 1.03, 95% CI 0.61 to 1.75) although the risk was higher than in those who only had busulfan. Other factors such as female sex and younger age were strong predictors of VOD development (102). Various studies have investigated the effect of glutathione S-transferase (GST) genetic polymorphisms on developing VOD, with mixed results (103, 104). These polymorphisms have been linked to busulfan PK and clearance in particular, with patients who have high GST-metabolizing capacity being at risk of developing VOD through rapid depletion of intracellular glutathione GSH (105). In the future biomarkers such as these polymorphisms may become part of routine practice in order to individualize dosing of chemotherapy agents for patients.

Fludarabine is a purine analogue which has become a less toxic but effective agent replacing cyclophosphamide in modern conditioning protocols. Ivaturi et al. prospectively studied the PK and pharmacodynamics (PD) of 133 children undergoing HSCT for a variety of disorders with a variety of conditioning regimens which all included fludarabine. Young age and renal impairment were associated with a higher AUC. In patients with malignant diseases, disease-free survival was highest 1 year after HSCT in subjects who had a systemic fludarabine plasma (f-ara-a) cumulative AUC greater than 15 mg*hour/L compared to patients with a cAUC less than 15 mg*hour/L (82.6% versus 52.8%, *p* = 0.04) (106). Other centers have also reported fludarabine PK studies and interest is growing due to its use for lymphodepletion in CAR T cell therapies (107–109). Further development of model-based dosing may reduce toxicity and increase efficacy, leading to superior outcomes for all patients.

Whilst studying the PK and PD of individual conditioning agents has provided information on possible targets for cumulative AUCs, combinations of different agents and their possible interactions as shown by Bognar et al. also has an impact on outcome and toxicities.

Patients with deoxyribonucleic acid (DNA)-repair disorders are at risk of toxicity from alkylating agents, and a reduced intensity regimen, for example, using low-dose cyclophosphamide and fludarabine is advisable (40, 110).

Alternative immune-based therapies, including antibody–drug conjugates, radio-labelled antibodies and CAR-T cells are being developed (111).

Monoclonal antibodies targeting CD117 are an attractive option. CD117 is the receptor for stem cell factor (SCF), a critical cytokine for HSC survival, maintenance and proliferation in the stem cell niche (112). If the receptor is blocked HSCs are deprived of SCF signalling, which leads to HSC depletion. A clinical trial in patients with SCID showed that the drug was well tolerated, with 4 of 6 patients 24 weeks post-HSCT showing successful engraftment (> 5% donor myeloid chimerism) and all having production of donor-derived T or B cells after 36 weeks (113). Combining antibodies with minimal intensity conventional chemotherapy agents such as an anti-CD45 antibody together with fludarabine and low-dose cyclophosphamide also resulted in successful engraftment (114).

Combinations of antibodies are being explored rather than using one single antibody to enhance engraftment. A further method is to combine an antibody with a drug – such conjugates combine a toxic payload to an antibody by a short linker molecule. This provides the combination of the toxicity of classic chemotherapy with the specificity of antibody mediated cell targeting. Saporin is a toxin that inactivates ribosomes but lacks a cell entry mechanism and it has been combined with anti-CD45 and studied in SCID mouse models (115, 116). Chimeric antigen receptor T (CAR-T) cells are an emerging immunotherapy in which the CAR is an engineered synthetic receptor designed to target a specific antigen (117). *In vivo* studies of a CD117 CAR-T cell showed the potential of CAR-Ts to be used in conditioning. In a CGD mouse model, a conditioning regimen of CAR-T with cyclophosphamide followed by HSCT allowed phenotypic correction of the disease (118).

Radioimmunotherapy is another alternative in which the cytotoxic effect of radiation can be delivered to the HSCs reducing the toxicity to other organs by conjugating radioisotopes to specific antibodies (119). For children, limited published data exists, but a conjugate of ^90^Yttrium to an antibody targeting CD66 was used in combination with melphalan and fludarabine or TBI for the treatment of children with significant comorbidities with malignant and non-malignant diseases. Fifteen of 16 children with non-malignant disease survived, 13/15 with complete donor chimerism. The 2 year disease-free survival was 94% (120).

Use of different serotherapy agents, dose and timing in relation to HSCT all have an impact on outcome of transplant in terms of occurrence of GVHD, level of donor chimerism and speed of immune reconstitution which affects risk of viral reactivation and clearance of infection. Marsh et al. reported that significantly higher levels of aGVHD, higher levels of donor chimerism, lymphocyte counts at D+30 and T cell counts at D+100 were associated with lower alemtuzumab levels at day 0 in 105 patients (121). They have gone on to suggest that an ideal therapeutic concentration window for day 0 alemtuzumab concentrations is 0.15–0.6 μg/mL and have reported a model-informed reduced alemtuzumab dosing with a target concentration intervention strategy which successfully increased the number of patients achieving this target concentration window (122).

There are 2 widely used types of anti-thymocyte globulin (ATG) (Fresenius/Grafalon and Genzyme) and the clearance of their active components differs. Oostenbrink reported results in 38 children with malignant hematological disorders. ATG Fresenius was cleared rapidly and uniformly from the circulation whether they received 60 mg/kg or 45 mg/kg, but there were significant differences in patients who received a high dose of ATG Genzyme (10 mg/kg) who had significantly slower reconstitution for CD3, CD4, and CD8 T cells compared to patients who received a low dose of ATG Genzyme (6–8 mg/kg) or ATG Fresenius (123). Admiraal et al. have recently reported the results of a prospective, single-arm, phase 2 clinical trial in which patients received intravenous ATG according to an individualized dosing nomogram which takes account of actual bodyweight, baseline absolute lymphocyte count, and graft source stem. Individualized dosing led to a significant improvement in early CD4^+^ immune reconstitution and no increases in incidence of GVHD and graft failure suggesting that this approach might improve outcomes of allogeneic HSCT (124).

In addition to conditioning, stem cell source plays an important part in influencing outcome from HSCT. Slatter et al. showed significantly higher myeloid chimerism in patients who received peripheral blood stem cells (PBSC) compared to cord blood and bone marrow. Higher CD34+ stem cell doses are given using PBSC. There was no significant association between acute or chronic GVHD and stem cell source (91).

In our center we choose PBSC in preference to bone marrow when using a MUD and we use Alemtuzumab in the conditioning regimen.

Key concepts in conditioning:

- Reduced toxicity regimens have improved outcome
- Further PK and PD studies of all agents used may further improve outcome
- Biomarkers may enable patient-tailored dosing in the future
- Antibody-based conditioning regimens may be the next big advancement
- Use of PBSC leads to higher myeloid chimerism

## T cell depletion

New methods of T cell depletion (TCD) have transformed our ability to successfully transplant all patients who are eligible for HSCT. Lack of a matched family or MUD are nolonger a barrier and outcomes from mismatched donor transplant are equivalent in some groups of patients to matched donor HSCT.

Graft manipulation by depleting GVHD-inducing CD3+ T cell receptor alpha beta cells reduces the risk of GVHD while retaining populations of gamma delta receptor T cells in the graft, which may contribute to early immune reconstitution and viral or tumour clearance (125–127).

An attractive alternative approach to *in vitro* T lymphocyte depletion is to infuse replete HLA-haplo-identical product followed by 2 doses of cyclophosphamide after transplant. Rapidly proliferating cells are preferentially targeted by the cyclophosphamide so alloreactive donor T lymphocytes are selectively deleted, which reduces the risk of GVHD, but retains viral specific T lymphocytes and lymphocyte precursor cells (128).

## CD3+TCR alpha beta/CD19+depletion

An increasing number of reports confirm the successful use of CD3+ TCR alpha beta/CD19+ depleted Haploidentical HSCT (129–135). A large series of 70 children affected by IEIs (40), inherited/acquired bone marrow failure syndromes (16), red blood cell disorders (11), or metabolic diseases (3), was recently reported from Rome. All received CD3+ TCR alpha beta/CD19+ depleted haploidentical HSCT from an HLA-partially matched relative. The median age at transplant was 3.5 years (range 0.3-16.1). Primary engraftment occurred in 51 patients, while 19 and 2 patients experienced primary or secondary graft failure (GF) respectively (30.4%). Most GFs were observed in children with diseases at risk for this complication such as aplastic anemia and thalassemia. All except 5 of these patients were successfully retransplanted. Six patients died of infectious complications (4 had active or recent infections at the time of HSCT), the cumulative incidence of TRM was 8.5%. The cumulative incidence of grade 1-2 acute GVHD was 14.4% and no patients developed grade 3-4 acute GVHD. Only 1 patient developed mild chronic GVHD. With a median follow-up of 3.5 years, the 5-year probability of overall and disease-free survival was 91.4% and 86.8%, respectively (136).

Lum et al. reported successive improvements in outcome over 30 years in 115 patients with IEI with changes in the method of T cell depletion. Thirty-four patients received CAMPATH-1M depleted bone marrow, 34 had CD34+ selected products, 7 CD3+/CD19+ PBSC and 40 CD3+ TCR alpha beta/CD19+ depleted PBSC. The 5-year OS was 58% (95% CI, 40–77%) for CAMPATH-1M, 68% (95% CI, 49–81%) for CD34+ selection, 69% (95% CI,23–92%) for CD3+/CD19+ depletion and 84% (95% CI, 67–93%, p=0.01) for CD3+ TCR alpha beta/CD19+ depletion (137). Conditioning changed from busulfan-based to reduced toxicity treosulfan-based and supportive care also improved with time. Nevertheless outcomes significantly improved and the indication for an ex-vivo T-cell depleted graft changed from predominantly SCID to non-SCID IEI which is remarkable given the poor historical outcomes from haploidentical HSCT for non-SCID IEI (11).

In our center in 41 conditioned transplants for SCID, 3-year OS was 91% for CD3+ TCR alpha beta/CD19+ depleted HSCT, 80% for MFD, 87% for MUD, and 89% for unrelated cord blood HSCT (p = 0.89). This demonstrated that the outcome of CD3+ TCR alpha beta/CD19+ depleted HSCT is comparable outcome to MUD HSCT and therefore is an attractive alternative donor strategy for infants with SCID who lack a matched donor (138).

Lum et al. also compared outcomes of 117 T-cell–replete HLA-matched grafts using alemtuzumab and 47 T-cell–depleted HLA-mismatched grafts using CD3+ TCR alpha beta/CD19+ depletion in children with IEI who received a first HSCT between 2014 and 2019. The 3-year OS for the whole cohort was 85%. The 3-year OS was comparable between MFD/MUD (88%) and CD3+ TCR alpha beta/CD19+ depleted HaploSCT (87%) in patients who were less than 5 years of age at transplant. However, for older children, more than 5 years of age, the OS was significantly lower in CD3+ TCR alpha beta/CD19+ depleted (55%), compared to MFD/MUD (87%) (p=0.03). This excess mortality was driven by viral infection and its associated complications. The cumulative incidence of viraemia at 6 months after transplant was significantly higher in CD3+ TCR alpha beta/CD19+ depleted haploSCT (80%) compared to MFD/MUD (55%) (p<0.001). The median day to CD3+ count of 200 cells/mL was 66 (range, 33-133 days) in T-replete graft patients and 89 days (range, 34-397 days) in T-depleted graft patients (p=.003) (139). Therefore further improvements need to be made to accelerate immune reconstitution and reduce the risk of viral infection particularly in this group of patients.

Additional donor T cells which have been genetically modified with a CaspaCIDe safety switch as a mechanism for inducing cell death, have been used post T-cell depleted HSCT. This provides a safety net to eliminate alloreactive modified T cells *via in vivo* administration of an activator agent, rimiducid if uncontrollable GvHD or other T-cell mediated transplant complications occur (140–142).

Memory (CD45RO+) T cells have the potential to confer pathogen-specific immunity early post-transplant but are sacrificed by CD3+ TCR alpha beta depletion techniques along with the CD45RA+ naive T cells which are effectors for GVHD. An alternative or additional strategy is to deplete CD45RA+ cells by a similar technique resulting in a cellular product passively enriched for CD45RO+ memory T cells. Early phase clinical trials have demonstrated the safety of this CD45RA-depletion approach: anti-viral efficacy and enhanced immune reconstitution have been demonstrated (143, 144). Thirty-six patients with malignant and 17 with non-malignant disorders were reported following donor lymphocytes depleted of CD45RA-positive cells in escalating doses post either haploidentical (n=25) or MUD(n=28) transplant. The OS rates were 80% and 88% in malignant and non-malignant conditions, respectively. In patients with no CMV-specific immune reactivity at baseline (n = 31) expansion of CMV-specific T-cells was demonstrated in 20 (64.5%) within 100 days (145). St Jude Children’s Research hospital have demonstrated an increase in the 3-year OS and EFS in non chemo-refractory recipients receiving CD45RA-depleted grafts (78.9% and 77.7%, respectively) compared with historic T-cell depleted haploHSCT cohorts (46.7% and 42.7%, respectively, p = 0.004, and 0.003). One hundred and forty-three paediatric and young adult patients with malignancy received a 1st allogeneic HSCT in 6 consecutive ex vivo T-cell depleted haploHSCT protocols over 15 years including 50 patients with CD45RA-depleted additional cells (146).

In a pilot project in our center 8 patients received an add-back of CD45RO+ memory T cells on day + 1 post CD3+TCR alpha beta/CD19+depleted mismatched HSCT. Numbers were small but there were fewer viraemias with CMV, EBV, Adenovirus and HHV6 compared to 42 historical patients who did not receive an add-back. Four patients had grade II skin GVHD which was easily controlled. Further work is planned to study the impact of ATG, timing and dose of add-back cells to give maximum benefit (147).

For a patient with SCID our practice is to choose a haploidentical donor if no matched family donor is available, thus avoiding the time and expense taken to look for an unrelated donor. For patients with non-SCID IEI we choose a haploidentical donor if no 10/10 HLA matched unrelated donor can be found and give an add-back of CD45RO+ cells in those with prior viral infection.

## Post transplant cyclophosphamide (PTCy)

In 2012 Bolanos-Meade et al. published results of a nonmyeloablative bone marrow transplantation platform using related, including HLA-haploidentical, donors for patients with sickle cell disease. The conditioning regimen consisted of ATG, fludarabine, cyclophosphamide, and TBI, with GVHD prophylaxis of post-transplantation high-dose cyclophosphamide, mycophenolate mofetil, and tacrolimus or sirolimus. They transplanted 17 patients, 14 from HLA-haploidentical and 3 from HLA-matched related donors. Eleven patients had durable engraftment. With a median follow-up of 711 days (minimal follow up 224 days), 10 patients were asymptomatic, and 6 patients off immunosupression. Only 1 patient developed skin-only acute GVHD that resolved without any therapy and there were no deaths (148). Rastogi et al. reported 8 patients with IEI including 3 with HLH with a variety of conditioning but all received 50mg/kg of cyclophosphamide post HSCT. Seven received haploidentical donors and 1 a MUD. Two children died: 1 with DOCK-8 deficiency with pretransplant infections died of sepsis on day+14; another child with HLH died due to thrombotic microangiopathy (TMA) leading to acute renal failure and death on day +28. OS was 75% at median follow up of 753 days (range, 248–1,222 days) (149). Uppuluri and colleagues reported 16 patients with IEI who received haploidentical HSCT; OS was 62.5% and grade II–IV aGvHD was 50% at a median follow-up of 23.3 months (150). Neven et al. have reported 27 patients with IEI who received haploidentical HSCT with an OS of 77% and grade II–IV aGVHD of 46% at a median follow-up of 25.6 months (151). Kurzay et al. reported 5 patients with IEI out of a total of 13 patients with non-malignant disorders. One patient with autosomal-dominant anhidrotic ectodermal dysplasia with immunodeficiency died and a patient with purine nucleoside phosphorylase deficiency required a second procedure. After a median follow up of 34.8 months OS was 92% and EFS was 77% (152). Mallhi and colleagues reported results of 23 patients using haploidentical HSCT. All patients had non-malignant disorders including 3 with SCID, 1 CGD and 5 with HLH. After a median follow-up of 2.5 years, OS and EFS were 91% and 78%, respectively. Grade II–IV aGVHD occurred in 78% (153). Olaya et al. reported 47 patients with IEI using different donors. Nineteen patients received T-replete haploidentical HSCT with PT Cy with an OS of 72% (154). Klein et al. reported 25 patients with IEI, PIRDs and inherited bone marrow failure syndromes with a PTCy approach. Donors were 14 haploidentical related, 9 MUD and 2 mismatched unrelated. With a median follow-up of 26 months (range 7 months–9 years), the 2-year OS was 92%. There were 2 deaths, 1 from infection, and 1 from complications after a second myeloablative HSCT. Three patients developed secondary graft failure, which were successfully treated with a CD34 cell boost in 1 or second HSCT in 2 cases (155).

There is no doubt that there is a place for both post transplant cyclophosphamide and CD3+TCR alpha beta depletion depending on center expertise. There is a retrospective IEWP EBMT study ongoing comparing the 2 platforms for patients with IEI and results are eagerly awaited.

Key concepts in TCD:

- *In vitro* TCD using CD3+TCR alpha beta/CD19+depletion or *in vivo* TCD using post transplant cyclophosphamide have revolutionized HSCT practice
- All patients eligible for HSCT now have a donor
- Further methods to accelerate immune reconstitution are needed to improve outcome

## Discussion

Significant advances have been made in the diagnosis and management of patients with IEIs undergoing HSCT. Many countries have adopted newborn screening for SCID in order to facilitate early diagnosis before infection occurs and thus enable prompt definitive cure with either HSCT or gene therapy if available. New challenges include the need for guidelines for keeping such patients free from infection, and practice varies in the time taken to, and method of HSCT between centers. The aim is to carry out procedures now that lead to robust immune reconstitution without toxicity, in order to ensure such infants will become adults who are not merely survivors, but have good quality of life. Such screening also picks up various other disorders apart from SCID, such as some babies born prematurely, thymic disorders and other T cell deficiencies, which all need to be managed (156, 157).

The genomic revolution has led to an exponential rise in the number of IEIs which can be treated with HSCT. Precise molecular genetic diagnosis informs clinicians, patients and families whether a disease might be amenable to HSCT. Population-based studies documenting the course of patients with specific diseases are extremely useful in helping patients and clinicians evaluate the risks of HSCT versus the risks of conservative management. There is increasing understanding in patients affected with some diseases, with effects beyond the hematopoietic compartment, of features that will not be cured by HSCT and may still cause significant morbidities in the future. Specific and targeted therapies are now available for some diseases which may enable patients to lead healthier lives without undertaking the risk of HSCT. The very long-term effects of these agents are however unknown and using them as bridging treatments to optimize patient status prior to HSCT is an attractive option.

Gene therapy has been successful for a number of diseases and usually requires lower doses of chemotherapy conditioning than HSCT, thus leading to fewer toxicities. There is a huge challenge however to make such treatment affordable and available (158, 159).

One of the major advances in leading to better outcomes from HSCT is the use of reduced toxicity conditioning and precise serotherapy regimens. This is being further refined by PK studies and research into using agents such as antibodies alone or in combination to avoid toxicity both in the short- and long-term.

Whilst TCD transplants for SCID have been carried out moderately successfully for many years, non-SCID IEIs had high rates of rejection with previous methods. The new platforms using either *in-vitro* TCD with CD3+ TCR alpha beta/CD19+ depletion or *in-vivo* depletion using post transplant cyclophosphamide have enabled HSCT for all eligible patients. Care needs to be taken when considering family donors who may be affected by a disease, without manifesting symptoms, or may be carriers who may not be suitable as donors. However these TCD techniques can be applied to mismatched unrelated donors if no family donor is available. Further improvements in immune reconstitution are emerging using additional cellular therapy. Combining these approaches with therapeutic-drug-monitored conditioning agents or antibody-based conditioning and precise graft content may enable a personalized approach to HSCT limiting graft failure and GVHD, short and long-term toxicity, and promoting robust long-term immune reconstitution and quality of life.

## Author contributions

All authors listed have made a substantial, direct, and intellectual contribution to the work and approved it for publication. 
